# Successes and Hurdles in Stem Cells Application and Production for Brain Transplantation

**DOI:** 10.3389/fnins.2019.01194

**Published:** 2019-11-19

**Authors:** Daniel Henriques, Ricardo Moreira, Jens Schwamborn, Luís Pereira de Almeida, Liliana S. Mendonça

**Affiliations:** ^1^Center for Neuroscience and Cell Biology, University of Coimbra, Coimbra, Portugal; ^2^Center for Innovative Biomedicine and Biotechnology, University of Coimbra, Coimbra, Portugal; ^3^Luxembourg Centre for Systems Biomedicine, University of Luxembourg, Esch-sur-Alzette, Luxembourg; ^4^Faculty of Pharmacy, University of Coimbra, Coimbra, Portugal

**Keywords:** stem cells transplantation, brain, neuronal integration and survival, adult brain plasticity, regulatory framework

## Abstract

Brain regenerative strategies through the transplantation of stem cells hold the potential to promote functional rescue of brain lesions caused either by trauma or neurodegenerative diseases. Most of the positive modulations fostered by stem cells are fueled by bystander effects, namely increase of neurotrophic factors levels and reduction of neuroinflammation. Nevertheless, the ultimate goal of cell therapies is to promote cell replacement. Therefore, the ability of stem cells to migrate and differentiate into neurons that later become integrated into the host neuronal network replacing the lost neurons has also been largely explored. However, as most of the preclinical studies demonstrate, there is a small functional integration of graft-derived neurons into host neuronal circuits. Thus, it is mandatory to better study the whole brain cell therapy approach in order to understand what should be better comprehended concerning graft-derived neuronal and glial cells migration and integration before we can expect these therapies to be ready as a viable solution for brain disorder treatment. Therefore, this review discusses the positive mechanisms triggered by cell transplantation into the brain, the limitations of adult brain plasticity that might interfere with the neuroregeneration process, as well as some strategies tested to overcome some of these limitations. It also considers the efforts that have been made by the regulatory authorities to lead to better standardization of preclinical and clinical studies in this field in order to reduce the heterogeneity of the obtained results.

## Introduction

In 1868, the German biologist Haeckel coined the term “stem cell” (Haeckel, 1868), proposing in Natural Creation Story (Natürliche Schöpfungsgeschichte) that each organism came from one cell. Since then, many authors have contributed to the growing knowledge of the stem cell research field. Dunn’s (1917) study is credited as the first clear evidence of successful transplantation of central nervous system (CNS) tissue into the brain of adult mammals, with clear survival of the transplanted neonatal cortex tissue (Dunn, 1917). Others followed her steps, providing more evidence of the successful introduction of new brain cells into the adult mammalian brain. Nevertheless, the prevailing dogma at the time that the adult brain is devoid of plasticity would impair a real paradigm change, and therefore the field of cell transplantation into the brain would have to wait until the 1970s and 1980s to be really launched (reviewed in Bjorklund and Stenevi, 1985; Dunnett, 2010).

A remarkable study in the field of neural cells transplantation has been the work conducted by Perlow et al. (1979), producing the first robust evidence of functional recovery upon rat fetal brain tissue implantation in rat adult brain whose dopaminergic input to the caudate had been destroyed. Additionally, in the same year, Beebe et al. (1979) demonstrated for the first time that transplants of embryonic brain tissue originate extensive axonal networks forming synaptic connections with the host brain. Other landmark studies were the improvements in motor function of patients with Parkinson’s disease (PD) observed by Lindvall et al. (1990), promoted by transplantation of grafts of fetal dopaminergic neurons (Lindvall et al., 1992), as well as the more recent observation that a patient with PD, 24 years upon being transplanted with human cells derived from embryonic ventral mesencephalon, presented graft-derived dopaminergic reinnervation of the putamen (Li et al., 2016).

Moreover, in the last decades important studies promoted the development of new sources of stem cells prone to be tested for human transplantation, such as the establishment of lines of human embryonic stem cells (ESC), and cell reprograming that culminated in the development of induced pluripotent stem cells (iPSC) and their derived cells (Gurdon, 1962; Gurdon et al., 1975; Davis et al., 1987; Thomson et al., 1998; Takahashi and Yamanaka, 2006).

These studies carried us to the concept of stem cell-based personalized medicines and to the possibility of generating potentially any type of cell from a specialized cell by reprograming it. Thus, presently, we must ask what should be the next steps to keep moving forward. Certainly, there is much to know about the stem cells’ potential and safety as regenerative approaches, but there is much more to know about the limitations caused by the restricted plasticity of the adult brain, hampering the migration and functional integration of enough new graft-derived neurons, and glial cells to promote strong neuroregeneration upon brain injury. Moreover, the standardization of preclinical and clinical studies enabling comparison of results obtained in different studies and triggering a faster development of cell-based therapies is also essential.

## Cell-Based Therapy for Brain Regeneration

**FIGURE 1:**
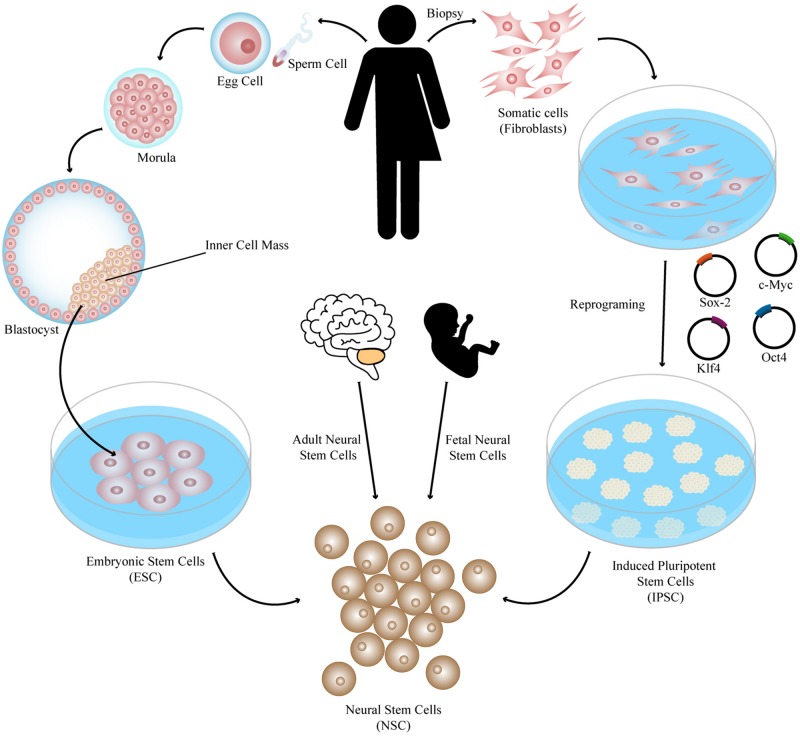
Different sources of stem cells to be used in brain regeneration. Pluripotent stem cells such as ESC obtained from the inner cell mass of the embryo’s blastocyst and iPSC obtained by cell reprograming of somatic cells by several protocols, such as expression of the reprograming factors Sox-2, Klf4, c-Myc, and Oct4, can be patterned and differentiated into different types of neural cells to be transplanted such as neural stem cells, which can also be isolated from the nervous system at different stages of development (fetal and adult neural stem cells).

Cell therapy consists of the use of cells or cell-based products in order to replace dead or defective cells with the purpose of restoring the tissue or organ functions lost in the disease or trauma process (Lindvall et al., 2004; Kim and de Vellis, 2009). There are different types of cells to be considered as a source of cells or as precursors of neural progenitors to be used in brain regeneration (Figure 1), namely, ESC obtained from the inner cell mass of the embryo’s blastocyst, iPSC obtained by cell reprograming, and neural stem cells that can be isolated from the nervous system at different stages of development, such as fetal and adult neural stem cells (Rippon and Bishop, 2004; Takahashi and Yamanaka, 2006; Kim and de Vellis, 2009). All of these cell types have strengths and drawbacks (Lo and Parham, 2009; Mendonca et al., 2018) and have been tested in different preclinical studies that proved them to be effective in the treatment of PD (Bjorklund et al., 2002; Wernig et al., 2008; Hargus et al., 2010), Huntington’s disease (HD) (Dunnett et al., 1998; Johann et al., 2007), and Machado-Joseph disease (MJD) (Mendonca et al., 2015). Importantly, some clinical trials have also demonstrated the great potential of these therapies in diseases like PD (Piccini et al., 1999; Olanow et al., 2003; Li et al., 2016; Bjorklund and Lindvall, 2017) and HD (Freeman et al., 2000). Despite the large number of preclinical studies and some clinical assays, describing positive results with cell therapy approaches for brain transplantation, the mechanisms behind such positive modulation as well as the types of cells promoting it are not fully understood.

### Understanding the Mechanism of Recovery Promoted by Stem Cell Transplantation

**FIGURE 2:**
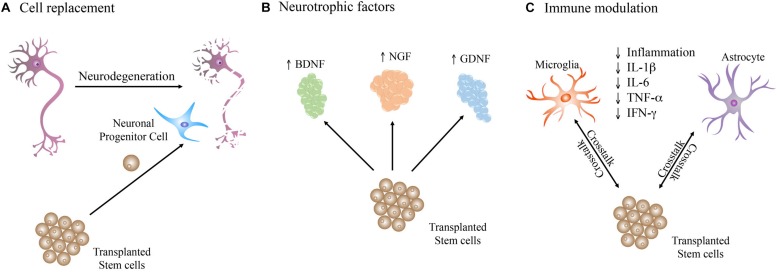
Therapeutic mechanisms triggered by stem cells upon transplantation in the diseased brain. Stem cells may act by **(A)** directly replacing the dead and impaired neurons in the neuronal network. **(B)** Production of neurotrophic factors that support the brain cells homeostasis. **(C)** Crosstalk with brain cells, such as astrocytes and microglia, which play important roles in immune regulation, leading to a reduction in inflammation through decrease of pro-inflammatory cytokines such as IL-1β, IL-6, TNF-α, and IFN-γ.

It has been described that the transplanted cells improve disease symptoms through the integration of new cells derived from the graft, provide trophic support to endogenous cells, and trigger immunomodulation (Figure 2A; Pluchino et al., 2005; Xu et al., 2011; Steinbeck and Studer, 2015). Nevertheless, the exact contribution of each of these positive mechanisms in the general improvements observed is unknown.

The functional integration of the graft-derived neurons into the host neuronal networks holds the potential to fully repair the damaged brain areas and rescue behavioral impairments. In fact, it has been suggested in different preclinical studies that the observed behavioral recovery is in part a result of the establishment of new synaptic connections between the brain and the graft (Clarke and Dunnett, 1993; Thompson et al., 2008; Cardoso et al., 2018), and clinical data support this evidence. In a remarkable study, cells derived from human embryonic ventral mesencephalon were transplanted in a PD patient, and 24 years upon the transplantation, authors observed graft-derived dopaminergic reinnervation of the putamen (Li et al., 2016). Nevertheless, most studies indicate that only a small number of graft-derived new neurons functionally integrate into the neuronal network (Cossetti et al., 2012; Forraz et al., 2013; Dunnett and Rosser, 2014). Additionally, given the described glia impairments in various conditions such as stroke, multiple sclerosis (MS), amyotrophic lateral sclerosis (ALS), PD, and Alzheimer’s disease (AD) (Miller et al., 2004; Lindvall and Kokaia, 2006; Dzamba et al., 2016; Kokaia et al., 2018), promoting the replacement of these cells has been tested and resulted in important positive outcomes (Windrem et al., 2004; Ericson et al., 2005) (described in more detail in section “Glial Cells Transplantation”).

Grafted cells are capable of increasing the survival and recovery of the host neurons by secreting neurotrophic factors such as brain-derived neurotrophic factor (BDNF), nerve growth factor (NGF), and glial cell-derived neurotrophic factor (GDNF) (Wang et al., 2013; Mendonca et al., 2015; Figure 2B), known to positively impact neural cells by promoting survival of host neurons, and survival, migration, and differentiation of the transplanted cells (Kamei et al., 2007; Xu et al., 2011; Ma et al., 2012).

The crosstalk between transplanted stem cells and the immune system (Figure 2C) in the brain is another important therapeutic mechanism (Kokaia et al., 2012). In fact, some studies demonstrated that transplanted stem cells decrease neuroinflammation, reducing neuronal death. The work of Pluchino et al. (2005, 2009) provided evidence that replacement of the affected or dead cells might not necessarily be the main mechanism behind the observed recovery upon stem cells transplantation, and it is instead the immune regulation that plays an important role in the observed improvements.

Thus, the transplantation of stem cells into the brain triggers several therapeutic mechanisms, and it would be of great importance to understand which cell type produces the positive effects, how these mechanisms are regulated, and to comprehend which features might be hindering better outcomes, namely in neuronal migration and integration in neuronal circuits of adult brain.

### Migration of Transplanted Cells

The brain is an organ with highly complex tissues composed of numerous different types of cells precisely organized. When damaged by trauma or disease, tissue regeneration involves complex processes. For instance, it is necessary that the transplanted cells be able to migrate to the affected areas in order to reach the proper and desired place and to be integrated (Fricker et al., 1999). The brain presents a limited capacity of structural repair after damage, not being able to fully recover from injuries. And in many cases, even if any amelioration of disease symptoms is visible, when the graft is analyzed it is perceived that transplanted cells into the adult brain tend to form clusters close to the site of transplantation demonstrating the low capacity of migration, thus limiting its regenerative capacity (Ladewig et al., 2014). To explain the lack of migration of the transplanted cells and also the low integration into the host brain, many several hypotheses have been suggested and investigated.

The maturity of the donor cells at the time of transplantation is an important aspect of the engraftment success. Different studies have achieved different rates of success when the transplanted cells presented different maturity. Ganat et al. (2012) used three mouse ESC cell lines, modified through the induction of specific cell stage transcription factors that mimic the *in vivo* progression of midbrain dopaminergic neuron development, namely early (Hes5), middle (Nurr1), and late (Pitx3) differentiation. These cells were transplanted into the striatum of adult unilateral 6-OHDA-lesioned immunocompromised mice, a PD mouse model. Authors observed that all cell lines, including the control cell line (parental cell line), originated robust tyrosine hydroxylase positive neurons. Nevertheless, the cell line corresponding to the earlier stage of development (Hes5) had a slightly lower yield than the other two cell lines. Nurr1 cells promoted more robust improvements on behavioral tests, indicating that cells in the middle stage of differentiation were ideal for ESC-derived dopaminergic neuron engraftment (Ganat et al., 2012). In a similar study performed by Payne et al. (2018), cortically specified neuroepithelial stem cells (cNESC) derived from iPSC were transplanted into a stroke-injured rat model 7 days post-injury, and transplantation success was analyzed 7 days later. Similarly to the previous study, the authors attempted to mimic three different stages of cell development. The cNESC were submitted to *in vitro* differentiation, promoted by the withdrawal of factors that maintained the immature state, plus BSA fraction V addition to the culture medium, establishing three different stages of cell maturation: early-differentiated cells at day 0, mid-differentiated at day 16, and late-differentiated stage at day 32 of differentiation. A higher number of graft-derived cells was observed in rats transplanted with the early and mid-differentiated cell groups. The higher number of cells observed was attributed to the survival of the initial transplanted population, demonstrating the importance of cell maturity for cell therapy success. Ladewig et al. (2014) also demonstrated that purified neurons presented increased migratory potential as opposed to neurons transplanted together with neural precursor cells. The authors found that factors such as FGF2 and VEGF expressed by neural progenitor cells, and not by mature neurons, acted as chemoattractants and were responsible for attracting neurons, reducing their migration. Authors demonstrated that chemoattraction inhibition through the pretreatment of cells to be transplanted with FGF2 and VEGF tyrosine kinase receptor inhibitor, the small molecule BIBF1120, or with neutralizing antibodies of FGF2 or receptor-blocking VEGF antibodies resulted in better migration. Furthermore, pretreated cells transplanted into the striatum of adult mice showed an increased extension of the graft, further spreading and generation of a less packed engraftment 1 week after transplantation (Ladewig et al., 2014).

Another hypothesis for the limited cell migration in the adult brain after transplantation lies in the differences between the developing and the adult brain. Looking at the nervous system dynamic composition during development, the role played by radial glial cells in this process is widely known. These cells are highly present during brain development but only a few persist in the adult brain (Barry et al., 2014) making them obvious targets of inquiry concerning possible altered processes in adult brain hindering cell migration. Briefly, the development of the CNS begins as an epithelial sheet that bends and forms the neural tube, composed by neuroepithelial cells, and then it expands at different rates to form the different areas of the CNS. Afterward, neuroepithelial cells change into radial glial cells retaining epithelial characteristics but becoming highly elongated. Radial glial cells then either directly generate neurons or other radial glial-like cells (intermediate progenitors and basal radial glia), which might later become neurons themselves. When neurons are produced in this stage, they migrate following the radial glia fascicles as a guide from their birthplace to their final locations. Radial glial cells ultimately differentiate into astrocytes and oligodendrocytes, and only a few remain in the adult brain in neurogenic niches (Merkle et al., 2004; Bonfanti and Peretto, 2007). Therefore, radial glial cells are crucial for the correct development of the brain and play a critical role on the migration of new neurons to their right locations (Nulty et al., 2015; Kelava and Lancaster, 2016). Besides the difference in the number of radial glial cells, the environment is also totally different (Gotz et al., 2016). During embryonic development, the surrounding environment promotes neurogenesis, inhibiting gliogenesis, while in the adult brain the predominant fate is gliogenesis (Kempermann et al., 2004; Miller and Gauthier, 2007). The impact of some extrinsic neurogenesis regulators change from the developing to the adult brain, for example, in the adult brain elements such as the blood-brain barrier, astrocytes, oligodendrocytes, and neuronal networks are elements that were absent during early development, which leads to different responses to extrinsic factors like neurotransmitters and growth factors by radial glial cells. This is the case for neurotransmitters like GABA and Glutamate that show opposing effects in the embryo and in the adult brain (Haydar et al., 2000; Nakamichi et al., 2009; Giachino et al., 2014; Gotz et al., 2016).

Other structures that play a role in the limited adult brain plasticity are the perineuronal nets (PNNs), described as a layer of lattice-like extracellular matrix aggregates surrounding the soma and proximal axons and dendrites of some neurons in different locations in the CNS such as the visual cortex (Pizzorusso et al., 2002), deep cerebellar nuclei (Carulli et al., 2006), substantia nigra (Bruckner et al., 2008), and hippocampus (Hylin et al., 2013). These PNNs are composed of different extracellular matrix molecules strongly present in the nervous system, namely chondroitin sulfate proteoglycans (CSPGs), hyaluronan, and link proteins (Kwok et al., 2011). These structures appear after neurodevelopment and are thought to contribute to the stabilization of synapses, limiting neuroplasticity. Nevertheless, PNNs can be modulated (in response to learning, stress or CNS injury/diseases), usually by the action of CSPG-degrading proteases (Lemarchant et al., 2016). A study has shown that degrading PNNs using chondroitinases ABC that remove chondroitin sulfate glycoaminoglycans (side chains attached to CSPG) can render the environment of the damaged CNS more permissive to axon regeneration (Moon et al., 2001). Thus, it is important to better understand the complete role of PNNs and if modulating its function might improve the migration and integration of newborn neurons after transplant.

The limited migratory capacity of the transplanted cells might be a result of a cocktail of conditions hindering cell migration, such as the inadequate maturation stage of the transplanted cells, the absence of guiding cues, and the presence of a supporting extracellular matrix in the adult brain that blocks cell migration. Thus, it is necessary to better comprehend the role played by these processes in the restricted migration that is usually observed in cell-based therapies and to exploit ways to mitigate these issues.

### Integration and Survival of New Neurons

The correct integration of graft-derived neurons into the host neuronal circuits is necessary to restore tissue functionality (Isacson et al., 1986; Steinbeck and Studer, 2015). Moreover, the transplanted cells must be capable of extending their neurites over long distances (Quadrato et al., 2014), which is quite demanding in the adult brain. Therefore, one aspect that has been addressed to increase graft integration is the axonal outgrowth, i.e., the projection of axons from the cell body to the target cells. This process is still present in the adult brain but is weakened as compared to the young brain and might contribute to the smaller regenerative capacity of the adult brain (Scheff et al., 1980; Ronn et al., 2000).

Therefore, several strategies have been tested to improve axonal outgrowths, such as genetically modifying the cells to be transplanted to overexpress described factors, exposure of donor cells to modulating compounds (Table 1) or making the host more prone to cell migration and axonal outgrowth (Steinbeck and Studer, 2015). Glaser et al. (2007) demonstrated that overexpression of polysialic acid (PSA) in embryonic stem cell-derived glial precursors enhances the cells’ sensitivity to migration guidance cues, and recent studies also tested the effect of PSA in axonal outgrowth. PSA is a carbohydrate expressed by neural precursors in both embryonic and adult brain (Hoffman and Edelman, 1983; Rutishauser and Landmesser, 1996) and has a major involvement in important steps of brain development, such as neural precursors migration, neuronal guidance, and synapse formation (Rothbard et al., 1982; Di Cristo et al., 2007; Jiang et al., 2016). Thus, its ability to reduce cell-to-cell interactions has been explored, which, consequently, promotes tissue plasticity (Battista et al., 2014). Battista et al. (2014) observed that the overexpression of the enzymes responsible for PSA synthesis, the polysialyltransferases (PST), resulting in higher PSA levels, led to an increase in axonal growth and enhanced behavioral recovery upon cell transplantation in a PD mouse model. In fact, ESC-derived dopaminergic neuron precursor cells overexpressing PST (Nurr1/PST) and control cells were transplanted into the striatum of 6-OHDA-lesioned mice. Control cells failed to produce any behavioral recovery, whereas with augmented PSA expression, the same number of cells produced significant recovery on PD motor impairments. Two months post-transplantation Nurr1/PST cells presented higher graft survival, more dopaminergic neuronal processes (dendrites and axons), increased axonal outgrowth, and increased synaptic marker synapsin that was correlated with functional recovery. Overall, these data demonstrate the crucial role of axonal outgrowth and graft-host innervation in the behavioral recovery.

**TABLE 1:** Targets and mechanisms of cell replacement modulation.

**Target**	**Strategy**	**Outcomes**	**References**
Neurotrophic factors (BDNF, NGF, and GDNF)	Expression of Neurotrophic factors by transplanted cells	Survival of host neurons, Survival, migration, and differentiation of transplanted cells	Kamei et al., 2007; Xu et al., 2011; Ma et al., 2012
Cell maturation	Transplant cells with the ideal maturity stage	Increase cell therapy success	Ganat et al., 2012; Payne et al., 2018
Neuroinflammation	Transplantation of cells to decrease neuroinflammation	Reduction of neuronal death	Pluchino et al., 2005, 2009
FGF2 and VEGF	Receptor inhibition; Neutralizing antibodies/receptor-blocking	Enhanced migration	Ladewig et al., 2014
Perineuronal nets (PNNs)	PNNs degradation with CSPGs^∗^-degrading proteases	Render CNS more permissive to axon regeneration	Moon et al., 2001; Lemarchant et al., 2016
Polysialic acid (PSA)	Increase PSA levels: Overexpression of PSA or Overexpression of the enzymes responsible for PSA synthesis	Increased axonal growth	Glaser et al., 2007; Battista et al., 2014
Myelin	Knockdown of Cdh1 to revert myelin associated inhibition of axonal growth	Increased axonal growth	Konishi et al., 2004; Poplawski et al., 2018

*^∗^CSPGs, chondroitin sulfate proteoglycans.*

Axonal growth in the CNS is often inhibited by molecules associated with adult myelin such as Nogo (Chen et al., 2000), myelin-associated glycoprotein (MAG) (McKerracher et al., 1994), and oligodendrocyte myelin glycoprotein (OMgp) (Kottis et al., 2002). Poplawski et al. (2018) recently investigated the interaction of adult myelin with axons extending from stem cell grafts. Plating different types of cells in different substrates, they confirmed that neurite growth from adult dorsal root ganglion (DRG) is inhibited by myelin and increased by laminin. Thus, different studies explored the potential of neutralizing these interactions in order to increase axonal outgrowth. Konishi et al. (2004) showed that inhibiting the anaphase-promoting complex (APC – highly expressed in postmitotic neurons and essential for cell cycle transition) by knockdown of Cdh1 (required for APC activity) enhanced axonal growth in primary cerebellar granule cells from postnatal day 6. Furthermore, they tested these cells’ ability to grow axons over myelin substrate. Here, axonal growth in control cells was significantly inhibited whereas the knockdown of Cdh1 reverted the myelin inhibition on axonal growth.

Lipid rafts have also been investigated when it comes to axonal growth and guidance. Lipid rafts are membrane microdomains enriched in cholesterol, glycosphingolipids and many proteins, namely proteins involved in cell signaling (Pike, 2003). As these lipid rafts are important for signaling transduction, they also play a role in neural development, namely the spatial and temporal control mediated by extracellular signals on axonal growth and guidance refereed by extracellular cues (Guirland et al., 2004; Kamiguchi, 2006; Guirland and Zheng, 2007). Age-associated loss of cholesterol in plasma membranes leads to loss of membrane lipid rafts, and consequently a decrease in its function (Egawa et al., 2016). Thus, it is important to investigate whether the age-related loss of membrane lipid rafts might play a critical role in axonal growth and guidance of the transplanted cells in the adult brain. Overall, different approaches have been tested to improve axonal elongation while trying to increase the success of cell-based therapies.

Besides the modulators assessed in this review (Table 1), several other molecules and mechanisms play an important role in neuronal migration and integration, such as neurotransmitters (Platel et al., 2010), neurotrophic factors (Waterhouse et al., 2012), and even pathological situations such as neuroinflammation (Whitney et al., 2009; Ryan and Nolan, 2016), which should also be taken into consideration.

### Glial Cells Transplantation

Glial cells are crucial for maintaining brain homeostasis, providing support and protection to neurons. Thus, cell therapies aiming at cell replacement in the brain, besides integration of new neurons into the neuronal network, should also consider promoting glial cells replacement, given the aforementioned impairment of these cells in several diseases (Miller et al., 2004; Lindvall and Kokaia, 2006; Dzamba et al., 2016; Kokaia et al., 2018) and also because upon transplantation glial cells promote brain functional recovery, namely by increasing axonal myelination, clearing aggregated proteins, reducing reactive oxygen species, and increasing survival, proliferation, and neural differentiation (Almad and Maragakis, 2012; Kokaia et al., 2018).

Brain trauma and diseases like MS, AD, PD, and leukodystrophy impair the production of new oligodendrocytes and their mediated axonal remyelination process (Bartzokis, 2004, 2011; Braak and Del Tredici, 2004; Franklin and Goldman, 2015). Windrem et al. (2004) showed that after oligodendrocyte progenitor cells transplantation into the forebrains of a leukodystrophy mouse model, the transplanted cells were capable of wide migration and differentiation into astrocytes and oligodendrocytes, showing increased myelin production and axonal myelination 12 weeks post-transplantation, resulting in phenotype improvement. Moreover, Piao and collaborators demonstrated that human ESC-derived oligodendrocytes upon transplantation into the brain of a rat model of radiation-induced demyelination are capable of migrating throughout white matter tracts, resulting in both structural and functional recovery (Piao et al., 2015).

In AD, astrocytes promote neuroprotection by reducing nitric oxide production mediated by microglia (Vincent et al., 1997) and by capturing and degrading Aβ plaques (Koistinaho et al., 2004). Nevertheless, the inability to continuously degrade these species confers aberrant and cytotoxic properties to astrocytes. Pihlaja and colleagues demonstrated that the transplantation of astrocytes into AD mice expressing human Aβ mediated a 70% reduction of Aβ plaques (Pihlaja et al., 2008) through proteolytic mechanisms (Pihlaja et al., 2011), demonstrating the ability of astrocytes transplantation to reduce the burden of Aβ plaques in the brain of AD mice. Moreover, the transplantation of glia restricted precursors (GRP) in the spinal cord of an ALS rat model, expressing human mutant SOD1, resulted in successful migration, differentiation, and integration of mature astrocytes. These increased the mice lifespan and survival of the motor neurons by neuroprotection mediated in part by the primary astrocyte glutamate transporter GLT1 (Lepore et al., 2008), demonstrating the potential therapeutic potential of astrocytes transplantation for ALS treatment. The transplantation of glial cell progenitors into spinal cord has also been shown to trigger neuroprotection in ALS by Haidet-Phillips and Maragakis (2015). Moreover, the co-transplantation of NSC with astrocytes into the ischemic striatum of a mouse model of stroke (middle cerebral artery occlusion) resulted in better outcomes when compared to transplantation of NSC alone, namely higher survival, proliferation, and neural differentiation of the transplanted NSC (Luo et al., 2017).

In a different approach, Thomsen et al. (2018) targeted cortical neurons through the transplantation of human cortical-derived neural progenitor cells engineered to secrete GDNF into the cortex of a transgenic mouse model of ALS (SOD1^*G93A*^). The transplanted cells migrated and differentiated into GDNF-releasing astrocytes, which resulted in motor neuron protection, delayed pathology, and extended animal lifespan. Ericson et al. (2005) have previously employed a similar strategy for PD treatment through the transplantation of rat astrocytes, transduced with lentivirus encoding for GDNF, into the striatum or substantia nigra of an adult rat model of PD. Results indicate that GDNF-expressing astrocytes maintained GDNF expression for 12 weeks and promoted neuroprotective effects on nigral tyrosine hydroxylase-positive cells. Moreover, Lundberg et al. (1996) also demonstrated that the transplantation of DOPA-secreting astrocytes into the striatum of a PD rat model (6-hydroxydopamine unilaterally lesioned rats) resulted in significant reduction of motor impairments 2 weeks post-transplantation, providing evidence of the therapeutic potential of *ex vivo* modifying glia to secrete L-DOPA, a keystone neurotransmitter in PD treatment, before transplantation.

Altogether, it has been demonstrated that the transplantation of glia and glial progenitors to improve the survival of other transplanted cells and trigger neuroprotective mechanisms has the potential to be used in the treatment of neurodegenerative diseases.

### Impact of Comorbidities in Stem Cell-Based Therapy Outcomes

Most preclinical studies using cell-based therapies have been performed without taking into account comorbidities, which might influence the outcomes of therapeutic strategies (Hermann et al., 2013; Sandu et al., 2015; Pradillo et al., 2017). Evidence indicates that neuroinflammation is one possible common pathway to several diseases, being fueled by conditions such as hypertension, diabetes, atherosclerosis, PD, HD, AD, and aging (Nguyen et al., 2002; Denes et al., 2012; Sandu et al., 2015). Aging is one of the most common risk factors for CNS-related diseases affecting brain function (Mattson and Arumugam, 2018). As the brain ages, several cellular and molecular functions become impaired (Niccoli and Partridge, 2012), leading to multiple modifications such as extensive neuronal loss, decreased neurotransmitters and their receptors levels, impaired myelination, reduced neurotrophic factors levels (Yang et al., 2012; Roozbehi et al., 2015), increased neuroinflammation (Currais, 2015; Deleidi et al., 2015), and decreased neurogenesis (Jinno, 2016) and brain plasticity (Badan et al., 2003). Considering that cell therapies applied to the treatment of brain diseases are frequently used to treat age-related diseases, such as neurodegenerative diseases with late-onset and stroke, aging effects in the therapeutic outcomes must be considered. As described above, the microenvironment of the aged brain is refractory for cell migration, proliferation, and differentiation, hindering the success of the transplanted cells (Della Porta et al., 2014; Conboy et al., 2015). Thus, in order for cell-based therapy preclinical tests to be clinically relevant, they must also be performed in aged animals so as to better predict the *in vivo* efficacy and capacity of the transplanted cells to promote beneficial effects (Popa-Wagner et al., 2014; Tatarishvili et al., 2014; Sandu et al., 2017).

### Impact of Combined Therapies in Stem Cell-Based Therapies Outcomes

Given that diseases are frequently multifactorial, combining different therapeutic strategies directed to different molecular and cellular targets may potently improve outcomes. Therefore, a promising therapeutic strategy is the combination of cell transplantation with drugs that act at comorbidities or enhance graft integration and corresponding brain regeneration. An interesting example is the combination of cell transplantation with the growth factor granulocyte colony-stimulating factor (G-CSF) that induces stem cell mobilization. Popa-Wagner et al. (2010) have shown that G-CSF administration after stroke in aged rats enhances neurogenesis and motor function parameters. Balseanu et al. (2014) demonstrated in a stroke rat model that daily intravenous injection of G-CSF led to robust and consistent improvement of neurological functions, which in combination with a single intravenous administration of mesenchymal stromal cells (MSC) resulted in significantly higher density of new blood vessels in the infarct core. However, the combination of G-CSF with bone marrow-derived mononuclear cells (BM MNC) also for stroke treatment led to no advantage over G-CSF treatment alone, suggesting that different outcomes might be achieved depending on the type of cells used (Buga et al., 2015). Sanchez-Ramos et al. (2009) tested G-CSF combination with human umbilical cord blood cell transplantation in a transgenic mouse model of AD and observed increased neurogenesis, reduced expression of amyloid proteins and inflammation, which resulted in an overall improvement of cognition in these animals. Similarly, another group tested the effect of G-CSF combined with human umbilical cord blood cells (hUCB) on traumatic brain injury (TBI), which reduced neuroinflammation and hippocampal cell loss, enhanced endogenous neurogenesis, and improved motor function, attaining better results than hUCB or G-CSF treatments alone (Acosta et al., 2014). Finally, the combination of statins, such as atorvastatin or simvastatin [promoting reduction of oxidative stress in the brain and clearing disease-causing proteins (Barone et al., 2011; Schultz et al., 2018)], with bone marrow stromal cells and MSC in a rat model of TBI, also resulted in increased cellular proliferation and differentiation, enhanced vascular density and neurological improvements, and behavioral amelioration (Mahmood et al., 2007, 2008).

Overall, the results obtained with combined therapies, including cell transplantation for brain regeneration and neuroprotection, indicate that better outcomes can be attained with this strategy.

## Standardization of Preclinical and Clinical Studies – The Regulatory Framework in the Particular Case of Cell-Based Therapies

The heterogeneous results obtained upon cell transplantation in some patients (Bachoud-Lévi et al., 2006; Cicchetti et al., 2009; Keene et al., 2009; Mendonca et al., 2018) and the increasing notion that the standardization of the procedures in cell transplantation field would accelerate development and guarantee the safety of new therapeutics (Bachoud-Lévi and Perrier, 2014) resulted in the establishment of several guidelines.

Cellular and gene therapies as well as tissue-engineered products, commonly known as advanced therapy medicinal products (ATMPs), are complex pharmaceutical products that represent a unique and challenging scientific and regulatory framework. Accordingly, to the regulatory framework scope, a cell-based medicinal product must contain viable human cells (of allogenic or autologous origins) that underwent a manufacturing process and may be combined with non-cellular components (such as scaffolds or matrixes) or be genetically modified (CHMP, 2008a). The major concern of regulators is to ensure the safety of these products for the patients, the population, and the environment, as well as to guarantee the efficacy of such treatments and their rapid entry into clinical practice to treat diseases that very often have no other therapeutic alternative (Lapteva et al., 2018).

The creation of regulations and guidance regarding ATMPs are under the responsibility of the European medicines agency (EMA) and the food and drug administration (FDA), in Europe and the United States, respectively. In particular, the advanced therapies are regulated by the committee for advanced therapies (CAT) and the office of cellular, tissue and gene therapies (OCTGT), from the EMA and FDA, respectively. Despite some differences, there are efforts for the harmonization of procedures between all markets under the international council for harmonization (ICH) of technical requirements for pharmaceuticals for human use, composed by the EMA, FDA, and the Japanese regulatory agency (Gee, 2018).

In this section, we review the processes and regulatory particularities regarding cellular and gene therapy from early manufacturing setting, to the definition of non-clinical profiling, and extrapolation to clinical assessments. The inclusion in this review of regulatory guidelines regarding gene therapy is justified by the common use of genetic modifications in cell-based products.

### Risk Evaluation

The complexity of these products makes the creation of a single regulatory strategy for the evaluation of risk difficult. Both the EMA and FDA recommend a risk-based approach and a case-by-case analysis for each product regarding their risk assessment. Not only the cellular nature but also the *ex vivo* manipulation, storage and shipment conditions, and pre-application procedures are a source of variability. Thus, EMA suggests that the risk analysis should cover the entire manufacture and non-clinical and clinical assessment of the product. The risk posed by cell-based therapies is dependent on the origin of the cells, the manufacturing process, the non-cellular components, and the therapeutic application, namely the action mechanism of the final product. The risk of cross-contamination of the product in any stage of manufacture is of utmost importance since it can threaten the quality of the final product. In addition, the environmental impact of the reagents used in cell culture must also be assessed (CHMP, 2008b; CAT/CPWP, 2013). The data presented when submitting a clinical trial application or a marketing authorization application must have in consideration the risks presented in risk management plan so that the safety of the product is assured (EMA, 2018).

From a clinical point of view, the main concern regarding cell-based medicinal products is the risk of tumor formation. Hence, cells must not be transplanted in a pluripotent state, which is associated with increased tumor formation. The *in vivo* microenvironment may also induce the cells to acquire tumor-like characteristics and, in turn, cells might induce host-derived tumors. The use of cells with short cell culturing periods is encouraged, given that cell manipulation may cause mutations responsible for tumor formation or loss of therapeutic potential. Moreover, the use of viral vectors for cell reprograming and gene correction might be responsible for the activation of oncogenes, depending on the site of gene insertion. The administration route of the cells is also crucial; intravenous administration is discouraged since cells can aggregate, causing emboli, or can accumulate in undesired peripheral organs, such as lungs, spleen or liver. The immune activation promoted by the cells is also a major issue that needs to be evaluated. Taken together, these represent risks associated with the use of cell-based products that strongly impact the success of these therapies (Herberts et al., 2011). The type of tests used to characterize the cells and their effects is a critical issue as well. For example, the establishment of tests for the evaluation of a cell’s potency, i.e., their biological activity, is particularly demanding given the frequent multiple action mechanisms of the cell-based products that might differ from product to product, and therefore regulatory entities adopted a flexible strategy. EMA, for example, allows the establishment of a product development adapted to its specificities, without a rigid framework with predefined requirements, and the FDA released technical recommendations although they are not mandatory (CBER, 2011; Pimpaneau et al., 2015).

### Manufacturing Process

It is often difficult to make the manufacturing process of cell-based medicinal products comply with the regulatory good manufacturing practices (GMP); nevertheless, these processes should be able to characterize, as full as possible, the product at several stages of manufacture and ensure product quality and yield at defined stages. The development of standard operating procedures (SOPs) is critical and the creation of in-process checkpoints to validate product quality and consistency is crucial (CBER, 2008; Carpenter, 2017; EC, 2017, 2018).

For example, in the manufacture of iPSC-derived products, the reprograming *per se* already represents a considerable manipulation of the cells, and it requires the use of manufacturing grade (instead of research grade) reagents that should also be used in the expansion and differentiation steps. The safety testing for infections, such as human immunodeficiency virus (HIV), of the starting materials is essential. For iPSC, the interclone variability after the reprograming should also be taken into consideration, and therefore the selection of the adequate clone for production is crucial since the reprograming could lead to severe karyotype abnormalities and mutations, which may interfere with the efficiency and safety of the final product. Thus, extensive characterization of phenotype, karyotype, and genotype of these cells is mandatory to ensure compliance with GMP. Furthermore, the expansion, cryopreservation, and differentiation protocols may also affect the quality, safety, and efficacy of the final product. Thus, valid biomarkers for each cell state are imperative; nevertheless, these biomarkers are not yet specified for any cell type used and strongly depend on a case-by-case analysis and on the desired characteristics of the final product (Carpenter, 2017; Stacey et al., 2018).

### Non-clinical Evaluation

**FIGURE 3:**
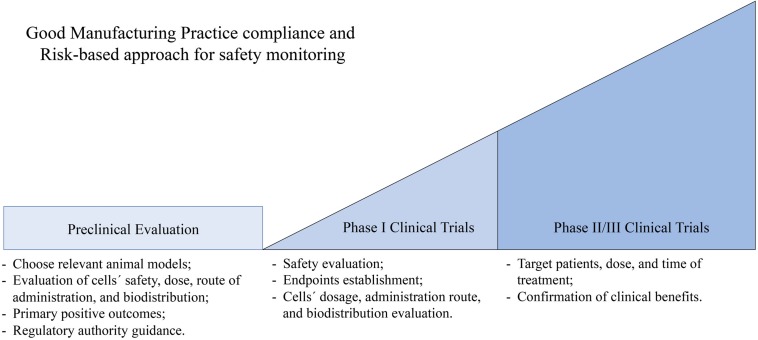
Illustration of some golden checkpoints in the preclinical and clinical evaluation process of cell-based therapies. At a preclinical level when testing cell-based therapies it is important to (i) choose a relevant disease model, (ii) assess cells’ safety, dose, biodistribution, and ideal route of administration, (iii) establish primary positive outcomes, and (iv) enter conversations with the regulatory authority. Considering phase I clinical trials, the key points are (i) safety evaluation, (ii) establishment of clearly defined endpoints, and (iii) cells’ dosage, administration route, and biodistribution evaluation. In phase II/III clinical trials it is important to establish (i) target patients, dose, and time of the treatment, (ii) clear primary and secondary endpoints, and (iii) sample size should provide statistical power to the endpoint evaluations.

Non-clinical evaluation of any medicinal product is a crucial step in their development to establish important clinical features such as pharmacodynamics, pharmacokinetics, or toxicology. These tests should be designed in order to establish the initial safe cell doses to be used in humans, the ideal route of administration, the biodistribution of the cells (accessing the organs more susceptible to the therapy/toxicity), and to assess potential side effects (CHMP, 2008a; CBER, 2013b; Figure 3).

The correct design of non-clinical testing for cellular therapies should start with the selection of a relevant animal model, which should mimic as closely as possible the pathology in humans, and should allow precise characterization of the treatment. The use of at least two different animal models, desirably from different species, is strongly encouraged. For a clear-cut assessment, animals from the Hominidae family may represent a good model to be used. Nevertheless, the use of the animal model must be clearly justified from a scientific point of view and the limitations of the use of such a model must be acknowledged (Salmikangas et al., 2015).

Another important aspect to be evaluated at this stage is the tumorigenicity of the cell therapy *in vivo*. The ability of the cells to migrate, uncontrollably grow or to differentiate should be defined. The non-clinical studies must also determine the long-term survival of the cells, their capacity to induce long-term toxicity, genotoxicity, and reproductive/developmental toxicity. In this regard, there are no specific recommended tests, and the analysis should depend on the product, its intent, and the risk management plan (Salmikangas et al., 2015).

Overall, this stage of testing of the cell-based medicinal product must, above all, indicate the safe use of this therapy in humans. Moreover, signs of its efficacy may be given, which can be demonstrated by the correct localization of the cells *in vivo*, their correct differentiation on the cell type desired, the improvement of the pathological characteristics of the disease (such as reduction of neuronal loss in neurodegenerative diseases), and by the alleviation of the animal’s phenotype (Salmikangas et al., 2015; Lapteva et al., 2018; Stacey et al., 2018).

The preclinical testing of a medicinal product may be quite costly. Thus, the initiation of an early conversation with regulatory authorities to seek their recommendation and input on the study design, as well as desired endpoint evaluations, becomes of the highest importance. Noteworthy is the fact that, at this point, the EMA and FDA have some regulatory differences. The EMA requires full compliance of GMP by the time the cell therapy is ready for Phase I human clinical trials. As for FDA regulations, their flexibility allows products not to be fully GMP compliant at early stage clinical trials (Carpenter, 2017).

### Clinical Studies

The complexity and invasive nature of cellular therapies define that all clinical testing must be performed in patients. The phase I trials should be designed taking into consideration the limitations of the preclinical tests performed and enroll a minimal number of patients sufficient to confirm the safety and efficacy. Standardization of all medical procedures, including administration route, dosage, and evaluation parameters, should be established at this stage and maintained throughout the clinical trial. Furthermore, the assessment of the cell’s behavior in the human body regarding its migration and differentiation capacity is also important. The results obtained at this stage must support and justify the testing of the medicinal product in a larger cohort of patients (CBER, 2013a; Salmikangas et al., 2015). Phase II/III clinical trials must clearly define the target population, dose and time of the treatment, the primary and secondary endpoints to be assessed during the clinical trial, and patient specificities, such as concomitant medication allowed. Moreover, the sample size must be sufficient to give statistical power to the observations and the endpoint evaluation enough to justify the attribution of marketing authorization by the regulatory authority (Salmikangas et al., 2015).

Several clinical trials using ESC- or iPSC-derived cells to treat a wide range of conditions are currently ongoing. Table 2 summarizes ongoing clinical trials using these types of cells in nervous system conditions. To this day, the approval of cellular therapies is somehow limited and, when granted, cases of withdrawal after short periods of time occurred (Carpenter, 2017).

**TABLE 2:** Clinical trials using pluripotent stem cells-derived cells in nervous system.

**CT**	**Condition/phase**	**Title**	**Type of cells**	**Sponsors and collaborators**
NCT03482050	Amyotrophic lateral sclerosis phase I/IIa	A study to evaluate transplantation of astrocytes derived from human embryonic stem cells in patients with amyotrophic lateral sclerosis (ALS)	AstroRx: Astrocytes derived from human embryonic stem cells	Kadimastem
NCT03119636	Parkinson’s disease phase I/II	Safety and efficacy study of human ESC-derived neural precursor cells in the treatment of Parkinson’s disease	Embryonic stem cells-derived neural precursor cells	Chinese Academy of Sciences
NCT02452723	Parkinson’s disease phase I	A study to evaluate the safety of neural stem cells in patients with Parkinson’s disease	Parthenogenetic neural stem cells^∗^	Cyto Therapeutics Pty Limited
NCT02302157	Spinal cord injury phase I/IIa	Dose escalation study of AST-OPC1 in spinal cord injury	Embryonic stem cell-derived oligodendrocyte progenitor cells	Asterias Biotherapeutics, Inc.

*CT, clinical trial number; ^∗^, derived from unfertilized oocytes; AST-OPC1, asterias -oligodendrocyte progenitor cell 1.*

## Conclusion

Presently, there are different types of stem cells and their derived neural progenitors prone to being tested in cell-based therapies for brain disorders treatment. Preclinical studies have demonstrated the undeniable potential of these cells to promote functional recovery of neurodegenerative diseases as well as other brain conditions through cell replacement and neuroprotection. Nevertheless, the integration of new functional neurons into adult neuronal circuits is a demanding and not yet fully understood process. A better comprehension of the modulating factors of the route taken by the graft-derived neurons during the migration, differentiation, and integration into the adult brain is of great importance in order to identify potential players and mechanisms that might be hindering a wider integration of new neurons and the restoration of neuronal circuits.

On the other hand, the higher complexity of cell-based products represents a challenge, since the manufacturing process becomes more difficult to standardize as compared with the classical chemical compounds or even with biologic products with more straightforward manufacturing processes and simpler therapeutic mechanisms. Nevertheless, the EMA and FDA have established a regulatory framework guiding procedures of preclinical and clinical assays to reduce heterogeneity, which is expected to enable comparison of results between studies.

Great hopes are set in cell-based therapies applied to neuroregenerative medicine, and although many questions are still to be answered these therapies have the potential to become the next generation of medicinal products.

## Author Contributions

All authors listed have made a substantial, direct and intellectual contribution to the work, and approved it for publication.

## Conflict of Interest

The authors declare that the research was conducted in the absence of any commercial or financial relationships that could be construed as a potential conflict of interest.
